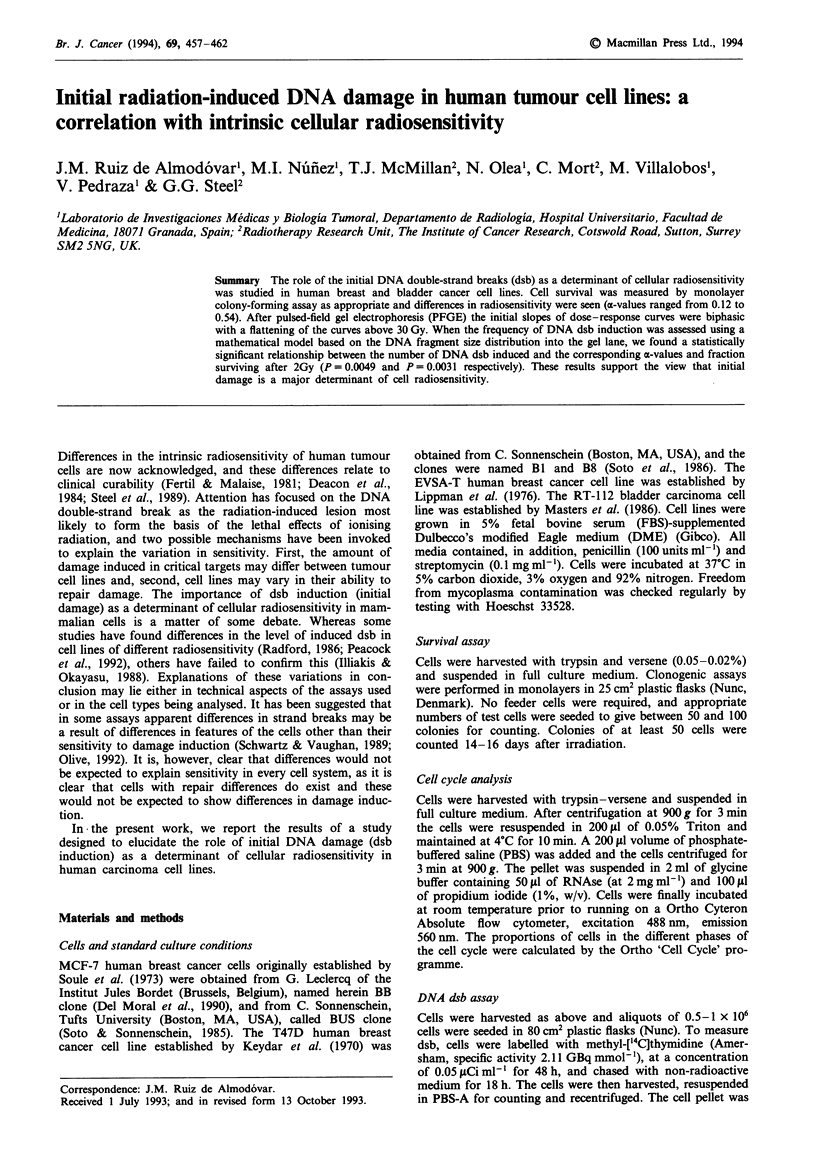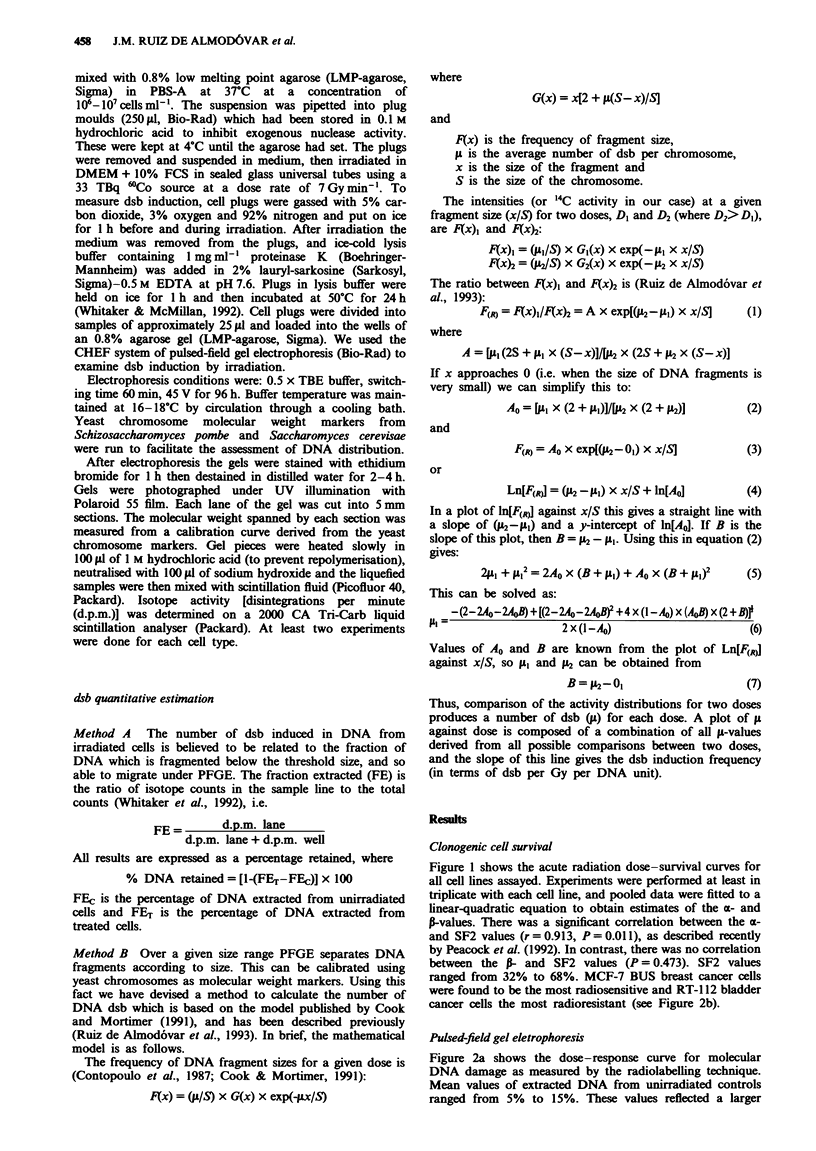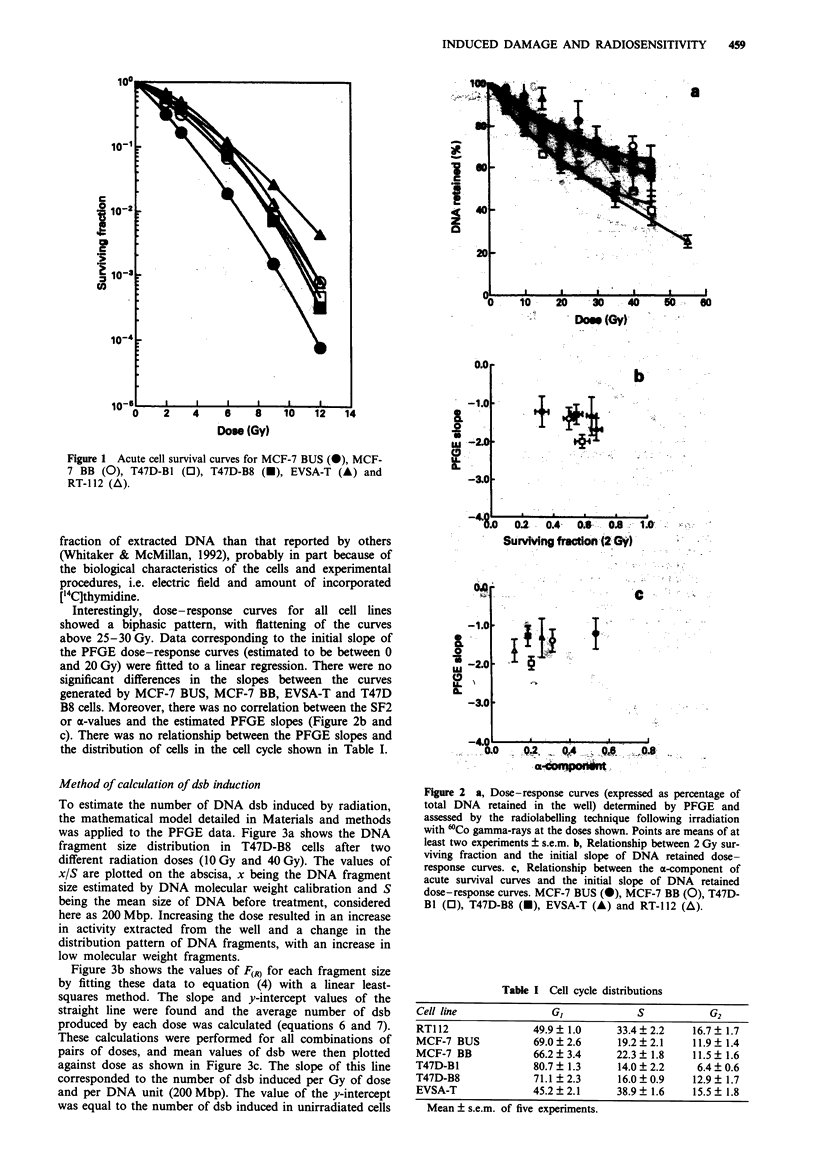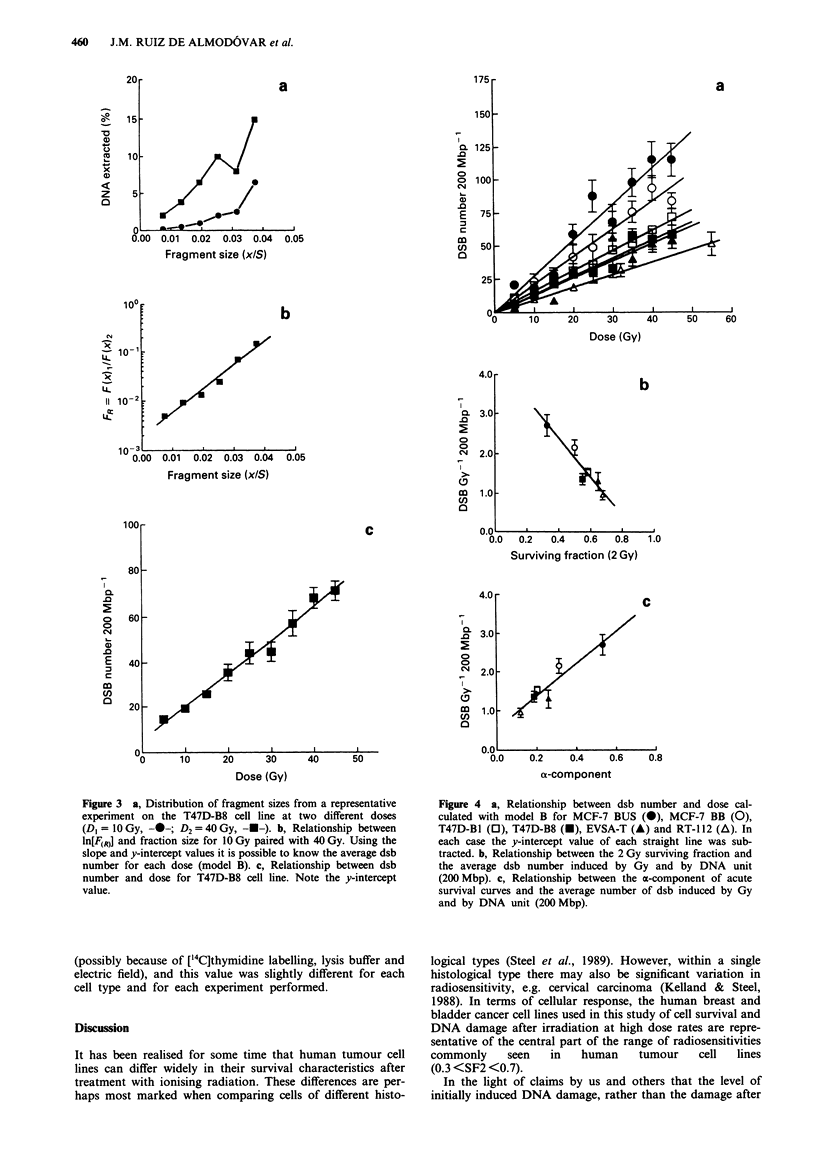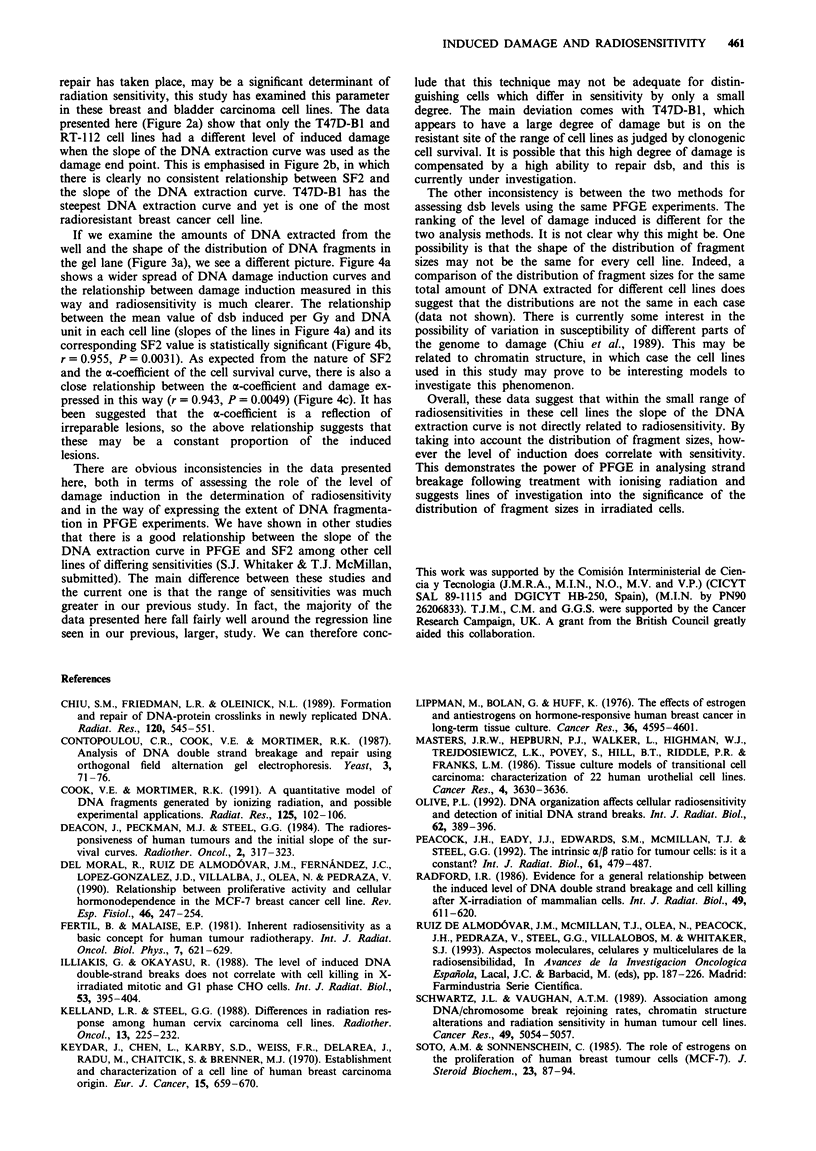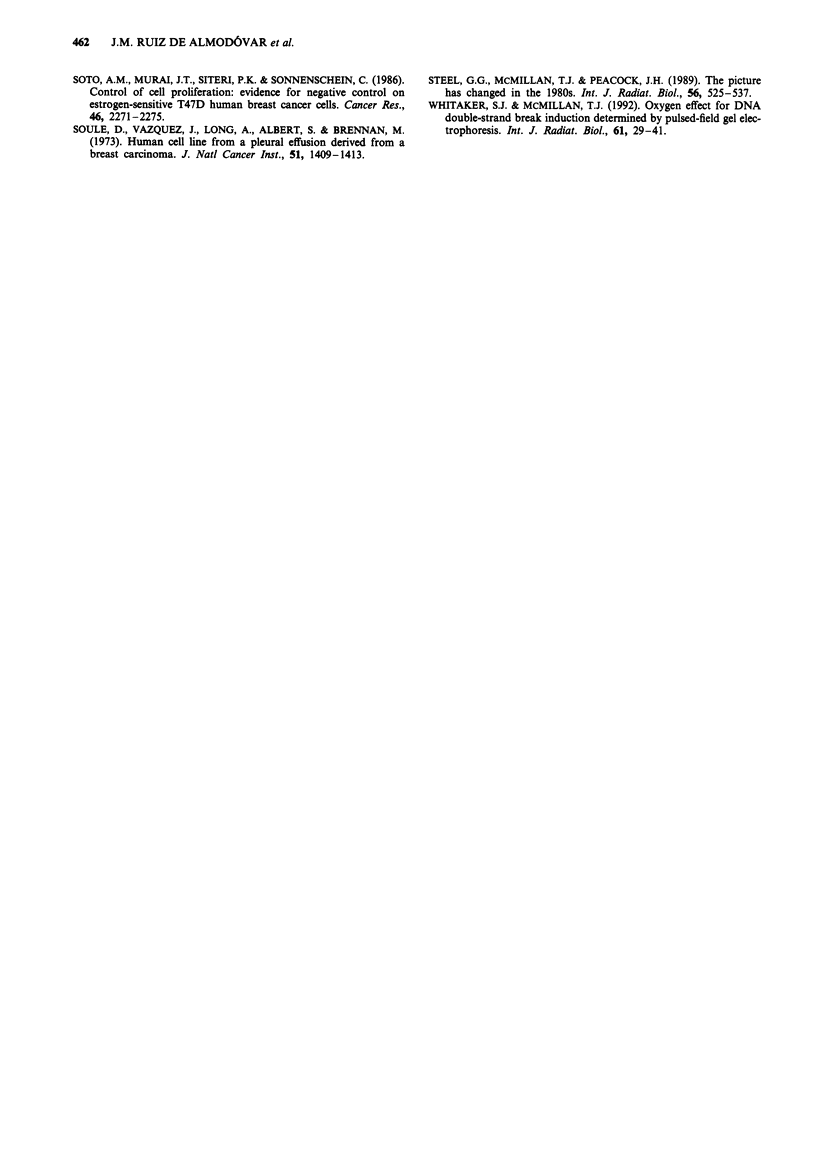# Initial radiation-induced DNA damage in human tumour cell lines: a correlation with intrinsic cellular radiosensitivity.

**DOI:** 10.1038/bjc.1994.83

**Published:** 1994-03

**Authors:** J. M. Ruiz de Almodóvar, M. I. Núñez, T. J. McMillan, N. Olea, C. Mort, M. Villalobos, V. Pedraza, G. G. Steel

**Affiliations:** Departamento de Radiologia, Hospital Universitario, Facultad de Medicina, Granada, Spain.

## Abstract

The role of the initial DNA double-strand breaks (dsb) as a determinant of cellular radiosensitivity was studied in human breast and bladder cancer cell lines. Cell survival was measured by monolayer colony-forming assay as appropriate and differences in radiosensitivity were seen (alpha-values ranged from 0.12 to 0.54). After pulsed-field gel electrophoresis (PFGE) the initial slopes of dose-response curves were biphasic with a flattening of the curves above 30 Gy. When the frequency of DNA dsb induction was assessed using a mathematical model based on the DNA fragment size distribution into the gel lane, we found a statistically significant relationship between the number of DNA dsb induced and the corresponding alpha-values and fraction surviving after 2Gy (P = 0.0049 and P = 0.0031 respectively). These results support the view that initial damage is a major determinant of cell radiosensitivity.


*Br. J. Cancer* (1994), **69**, 457–462 © Macmillan Press Ltd., 1994

### Initial radiation-induced DNA damage in human tumour cell lines: a correlation with intrinsic cellular radiosensitivity

J.M. Ruiz de Almodóvar[1], M.I. Núñez[1], T.J. McMillan[2], N. Olea[1], C. Mort[2], M. Villalobos[1], V. Pedraza[1] & G.G. Steel[2]

[1]*Laboratorio de Investigaciones Médicas y Biología Tumoral, Departamento de Radiología, Hospital Universitario, Facultad de Medicina, 18071 Granada, Spain;* [2]*Radiotherapy Research Unit, The Institute of Cancer Research, Cotswold Road, Sutton, Surrey SM2 5NG, UK.*

**Summary** The role of the initial DNA double-strand breaks (dsb) as a determinant of cellular radiosensitivity was studied in human breast and bladder cancer cell lines. Cell survival was measured by monolayer colony-forming assay as appropriate and differences in radiosensitivity were seen (α-values ranged from 0.12 to 0.54). After pulsed-field gel electrophoresis (PFGE) the initial slopes of dose–response curves were biphasic with a flattening of the curves above 30 Gy. When the frequency of DNA dsb induction was assessed using a mathematical model based on the DNA fragment size distribution into the gel lane, we found a statistically significant relationship between the number of DNA dsb induced and the corresponding α-values and fraction surviving after 2Gy ($P = 0.0049$ and $P = 0.0031$ respectively). These results support the view that initial damage is a major determinant of cell radiosensitivity.

Differences in the intrinsic radiosensitivity of human tumour cells are now acknowledged, and these differences relate to clinical curability (Fertil & Malaise, 1981; Deacon *et al.*, 1984; Steel *et al.*, 1989). Attention has focused on the DNA double-strand break as the radiation-induced lesion most likely to form the basis of the lethal effects of ionising radiation, and two possible mechanisms have been invoked to explain the variation in sensitivity. First, the amount of damage induced in critical targets may differ between tumour cell lines and, second, cell lines may vary in their ability to repair damage. The importance of dsb induction (initial damage) as a determinant of cellular radiosensitivity in mammalian cells is a matter of some debate. Whereas some studies have found differences in the level of induced dsb in cell lines of different radiosensitivity (Radford, 1986; Peacock *et al.*, 1992), others have failed to confirm this (Illiakis & Okayasu, 1988). Explanations of these variations in conclusion may lie either in technical aspects of the assays used or in the cell types being analysed. It has been suggested that in some assays apparent differences in strand breaks may be a result of differences in features of the cells other than their sensitivity to damage induction (Schwartz & Vaughan, 1989; Olive, 1992). It is, however, clear that differences would not be expected to explain sensitivity in every cell system, as it is clear that cells with repair differences do exist and these would not be expected to show differences in damage induction.

In the present work, we report the results of a study designed to elucidate the role of initial DNA damage (dsb induction) as a determinant of cellular radiosensitivity in human carcinoma cell lines.

### Materials and methods

### *Cells and standard culture conditions*

MCF-7 human breast cancer cells originally established by Soule *et al.* (1973) were obtained from G. Leclercq of the Institut Jules Bordet (Brussels, Belgium), named herein BB clone (Del Moral *et al.*, 1990), and from C. Sonnenschein, Tufts University (Boston, MA, USA), called BUS clone (Soto & Sonnenschein, 1985). The T47D human breast cancer cell line established by Keydar *et al.* (1970) was obtained from C. Sonnenschein (Boston, MA, USA), and the clones were named B1 and B8 (Soto *et al.*, 1986). The EVSA-T human breast cancer cell line was established by Lippman *et al.* (1976). The RT-112 bladder carcinoma cell line was established by Masters *et al.* (1986). Cell lines were grown in 5% fetal bovine serum (FBS)-supplemented Dulbecco's modified Eagle medium (DME) (Gibco). All media contained, in addition, penicillin (100 units $ml^{-1}$) and streptomycin (0.1 mg $ml^{-1}$). Cells were incubated at 37°C in 5% carbon dioxide, 3% oxygen and 92% nitrogen. Freedom from mycoplasma contamination was checked regularly by testing with Hoeschst 33528.

### *Survival assay*

Cells were harvested with trypsin and versene (0.05–0.02%) and suspended in full culture medium. Clonogenic assays were performed in monolayers in 25 $cm^2$ plastic flasks (Nunc, Denmark). No feeder cells were required, and appropriate numbers of test cells were seeded to give between 50 and 100 colonies for counting. Colonies of at least 50 cells were counted 14–16 days after irradiation.

### *Cell cycle analysis*

Cells were harvested with trypsin–versene and suspended in full culture medium. After centrifugation at 900 *g* for 3 min the cells were resuspended in 200 µl of 0.05% Triton and maintained at 4°C for 10 min. A 200 µl volume of phosphate-buffered saline (PBS) was added and the cells centrifuged for 3 min at 900 *g*. The pellet was suspended in 2 ml of glycine buffer containing 50 µl of RNAse (at 2 mg $ml^{-1}$) and 100 µl of propidium iodide (1%, w/v). Cells were finally incubated at room temperature prior to running on a Ortho Cyteron Absolute flow cytometer, excitation 488 nm, emission 560 nm. The proportions of cells in the different phases of the cell cycle were calculated by the Ortho 'Cell Cycle' programme.

### *DNA dsb assay*

Cells were harvested as above and aliquots of $0.5-1 \times 10^6$ cells were seeded in 80 $cm^2$ plastic flasks (Nunc). To measure dsb, cells were labelled with methyl-[$^{14}$C]thymidine (Amersham, specific activity 2.11 GBq $mmol^{-1}$), at a concentration of 0.05 µCi $ml^{-1}$ for 48 h, and chased with non-radioactive medium for 18 h. The cells were then harvested, resuspended in PBS-A for counting and recentrifuged. The cell pellet was

Correspondence: J.M. Ruiz de Almodóvar.
Received 1 July 1993; and in revised form 13 October 1993.

mixed with 0.8% low melting point agarose (LMP-agarose, Sigma) in PBS-A at 37°C at a concentration of $10^6-10^7$ cells $ml^{-1}$. The suspension was pipetted into plug moulds (250 μl, Bio-Rad) which had been stored in 0.1 M hydrochloric acid to inhibit exogenous nuclease activity. These were kept at 4°C until the agarose had set. The plugs were removed and suspended in medium, then irradiated in DMEM + 10% FCS in sealed glass universal tubes using a 33 TBq $^{60}Co$ source at a dose rate of 7 Gy $min^{-1}$. To measure dsb induction, cell plugs were gassed with 5% carbon dioxide, 3% oxygen and 92% nitrogen and put on ice for 1 h before and during irradiation. After irradiation the medium was removed from the plugs, and ice-cold lysis buffer containing 1 mg $ml^{-1}$ proteinase K (Boehringer-Mannheim) was added in 2% lauryl-sarkosine (Sarkosyl, Sigma)–0.5 M EDTA at pH 7.6. Plugs in lysis buffer were held on ice for 1 h and then incubated at 50°C for 24 h (Whitaker & McMillan, 1992). Cell plugs were divided into samples of approximately 25 μl and loaded into the wells of an 0.8% agarose gel (LMP-agarose, Sigma). We used the CHEF system of pulsed-field gel electrophoresis (Bio-Rad) to examine dsb induction by irradiation.

Electrophoresis conditions were: 0.5 × TBE buffer, switching time 60 min, 45 V for 96 h. Buffer temperature was maintained at 16–18°C by circulation through a cooling bath. Yeast chromosome molecular weight markers from *Schizosaccharomyces pombe* and *Saccharomyces cerevisae* were run to facilitate the assessment of DNA distribution.

After electrophoresis the gels were stained with ethidium bromide for 1 h then destained in distilled water for 2–4 h. Gels were photographed under UV illumination with Polaroid 55 film. Each lane of the gel was cut into 5 mm sections. The molecular weight spanned by each section was measured from a calibration curve derived from the yeast chromosome markers. Gel pieces were heated slowly in 100 μl of 1 M hydrochloric acid (to prevent repolymerisation), neutralised with 100 μl of sodium hydroxide and the liquefied samples were then mixed with scintillation fluid (Picofluor 40, Packard). Isotope activity [disintegrations per minute (d.p.m.)] was determined on a 2000 CA Tri-Carb liquid scintillation analyser (Packard). At least two experiments were done for each cell type.

*dsb quantitative estimation*

*Method A* The number of dsb induced in DNA from irradiated cells is believed to be related to the fraction of DNA which is fragmented below the threshold size, and so able to migrate under PFGE. The fraction extracted (FE) is the ratio of isotope counts in the sample line to the total counts (Whitaker *et al.*, 1992), i.e.

$$FE = \frac{\text{d.p.m. lane}}{\text{d.p.m. lane} + \text{d.p.m. well}}$$

All results are expressed as a percentage retained, where

$$\%\ \text{DNA retained} = [1\text{-}(FE_T - FE_C)] \times 100$$

$FE_C$ is the percentage of DNA extracted from unirradiated cells and $FE_T$ is the percentage of DNA extracted from treated cells.

*Method B* Over a given size range PFGE separates DNA fragments according to size. This can be calibrated using yeast chromosomes as molecular weight markers. Using this fact we have devised a method to calculate the number of DNA dsb which is based on the model published by Cook and Mortimer (1991), and has been described previously (Ruiz de Almodóvar *et al.*, 1993). In brief, the mathematical model is as follows.

The frequency of DNA fragment sizes for a given dose is (Contopoulo *et al.*, 1987; Cook & Mortimer, 1991):

$$F(x) = (\mu/S) \times G(x) \times \exp(-\mu x/S)$$

where

$$G(x) = x[2 + \mu(S-x)/S]$$

and

$F(x)$ is the frequency of fragment size,
$\mu$ is the average number of dsb per chromosome,
$x$ is the size of the fragment and
$S$ is the size of the chromosome.

The intensities (or $^{14}C$ activity in our case) at a given fragment size ($x/S$) for two doses, $D_1$ and $D_2$ (where $D_2 > D_1$), are $F(x)_1$ and $F(x)_2$:

$$F(x)_1 = (\mu_1/S) \times G_1(x) \times \exp(-\mu_1 \times x/S)$$
$$F(x)_2 = (\mu_2/S) \times G_2(x) \times \exp(-\mu_2 \times x/S)$$

The ratio between $F(x)_1$ and $F(x)_2$ is (Ruiz de Almodóvar *et al.*, 1993):

$$F_{(R)} = F(x)_1/F(x)_2 = A \times \exp[(\mu_2 - \mu_1) \times x/S] \quad (1)$$

where

$$A = [\mu_1 (2S + \mu_1 \times (S-x)]/[\mu_2 \times (2S + \mu_2 \times (S-x)]$$

If $x$ approaches 0 (i.e. when the size of DNA fragments is very small) we can simplify this to:

$$A_0 = [\mu_1 \times (2 + \mu_1)]/[\mu_2 \times (2 + \mu_2)] \quad (2)$$

and

$$F_{(R)} = A_0 \times \exp[(\mu_2 - 0_1) \times x/S] \quad (3)$$

or

$$\text{Ln}[F_{(R)}] = (\mu_2 - \mu_1) \times x/S + \ln[A_0] \quad (4)$$

In a plot of $\ln[F_{(R)}]$ against $x/S$ this gives a straight line with a slope of $(\mu_2 - \mu_1)$ and a $y$-intercept of $\ln[A_0]$. If $B$ is the slope of this plot, then $B = \mu_2 - \mu_1$. Using this in equation (2) gives:

$$2\mu_1 + \mu_1^2 = 2A_0 \times (B + \mu_1) + A_0 \times (B + \mu_1)^2 \quad (5)$$

This can be solved as:

$$\mu_1 = \frac{-(2-2A_0-2A_0B) + [(2-2A_0-2A_0B)^2 + 4 \times (1-A_0) \times (A_0B) \times (2+B)]^{\frac{1}{2}}}{2 \times (1-A_0)} \quad (6)$$

Values of $A_0$ and $B$ are known from the plot of $\text{Ln}[F_{(R)}]$ against $x/S$, so $\mu_1$ and $\mu_2$ can be obtained from

$$B = \mu_2 - 0_1 \quad (7)$$

Thus, comparison of the activity distributions for two doses produces a number of dsb ($\mu$) for each dose. A plot of $\mu$ against dose is composed of a combination of all $\mu$-values derived from all possible comparisons between two doses, and the slope of this line gives the dsb induction frequency (in terms of dsb per Gy per DNA unit).

### Results

*Clonogenic cell survival*

Figure 1 shows the acute radiation dose–survival curves for all cell lines assayed. Experiments were performed at least in triplicate with each cell line, and pooled data were fitted to a linear-quadratic equation to obtain estimates of the α- and β-values. There was a significant correlation between the α- and SF2 values ($r = 0.913$, $P = 0.011$), as described recently by Peacock *et al.* (1992). In contrast, there was no correlation between the β- and SF2 values ($P = 0.473$). SF2 values ranged from 32% to 68%. MCF-7 BUS breast cancer cells were found to be the most radiosensitive and RT-112 bladder cancer cells the most radioresistant (see Figure 2b).

*Pulsed-field gel eletrophoresis*

Figure 2a shows the dose–response curve for molecular DNA damage as measured by the radiolabelling technique. Mean values of extracted DNA from unirradiated controls ranged from 5% to 15%. These values reflected a larger

**Figure 1** Acute cell survival curves for MCF-7 BUS (●), MCF-7 BB (○), T47D-B1 (□), T47D-B8 (■), EVSA-T (▲) and RT-112 (Δ).

fraction of extracted DNA than that reported by others (Whitaker & McMillan, 1992), probably in part because of the biological characteristics of the cells and experimental procedures, i.e. electric field and amount of incorporated [$^{14}$C]thymidine.

Interestingly, dose-response curves for all cell lines showed a biphasic pattern, with flattening of the curves above 25–30 Gy. Data corresponding to the initial slope of the PFGE dose-response curves (estimated to be between 0 and 20 Gy) were fitted to a linear regression. There were no significant differences in the slopes between the curves generated by MCF-7 BUS, MCF-7 BB, EVSA-T and T47D B8 cells. Moreover, there was no correlation between the SF2 or α-values and the estimated PFGE slopes (Figure 2b and c). There was no relationship between the PFGE slopes and the distribution of cells in the cell cycle shown in Table I.

*Method of calculation of dsb induction*

To estimate the number of DNA dsb induced by radiation, the mathematical model detailed in Materials and methods was applied to the PFGE data. Figure 3a shows the DNA fragment size distribution in T47D-B8 cells after two different radiation doses (10 Gy and 40 Gy). The values of $x/S$ are plotted on the abscisa, $x$ being the DNA fragment size estimated by DNA molecular weight calibration and $S$ being the mean size of DNA before treatment, considered here as 200 Mbp. Increasing the dose resulted in an increase in activity extracted from the well and a change in the distribution pattern of DNA fragments, with an increase in low molecular weight fragments.

Figure 3b shows the values of $F_{(R)}$ for each fragment size by fitting these data to equation (4) with a linear least-squares method. The slope and $y$-intercept values of the straight line were found and the average number of dsb produced by each dose was calculated (equations 6 and 7). These calculations were performed for all combinations of pairs of doses, and mean values of dsb were then plotted against dose as shown in Figure 3c. The slope of this line corresponded to the number of dsb induced per Gy of dose and per DNA unit (200 Mbp). The value of the $y$-intercept was equal to the number of dsb induced in unirradiated cells

**Figure 2** **a**, Dose-response curves (expressed as percentage of total DNA retained in the well) determined by PFGE and assessed by the radiolabelling technique following irradiation with $^{60}$Co gamma-rays at the doses shown. Points are means of at least two experiments ± s.e.m. **b**, Relationship between 2 Gy surviving fraction and the initial slope of DNA retained dose-response curves. **c**, Relationship between the α-component of acute survival curves and the initial slope of DNA retained dose-response curves. MCF-7 BUS (●), MCF-7 BB (○), T47D-B1 (□), T47D-B8 (■), EVSA-T (▲) and RT-112 (Δ).

**Table I** Cell cycle distributions

| *Cell line* | $G_1$ | $S$ | $G_2$ |
|---|---|---|---|
| RT112 | 49.9 ± 1.0 | 33.4 ± 2.2 | 16.7 ± 1.7 |
| MCF-7 BUS | 69.0 ± 2.6 | 19.2 ± 2.1 | 11.9 ± 1.4 |
| MCF-7 BB | 66.2 ± 3.4 | 22.3 ± 1.8 | 11.5 ± 1.6 |
| T47D-B1 | 80.7 ± 1.3 | 14.0 ± 2.2 | 6.4 ± 0.6 |
| T47D-B8 | 71.1 ± 2.3 | 16.0 ± 0.9 | 12.9 ± 1.7 |
| EVSA-T | 45.2 ± 2.1 | 38.9 ± 1.6 | 15.5 ± 1.8 |

Mean ± s.e.m. of five experiments.

Figure 3 a, Distribution of fragment sizes from a representative experiment on the T47D-B8 cell line at two different doses ($D_1$ = 10 Gy, -●-; $D_2$ = 40 Gy, -■-). b, Relationship between ln[$F_{(R)}$] and fraction size for 10 Gy paired with 40 Gy. Using the slope and y-intercept values it is possible to know the average dsb number for each dose (model B). c, Relationship between dsb number and dose for T47D-B8 cell line. Note the y-intercept value.

Figure 4 a, Relationship between dsb number and dose calculated with model B for MCF-7 BUS (●), MCF-7 BB (○), T47D-B1 (□), T47D-B8 (■), EVSA-T (▲) and RT-112 (△). In each case the y-intercept value of each straight line was subtracted. b, Relationship between the 2 Gy surviving fraction and the average dsb number induced by Gy and by DNA unit (200 Mbp). c, Relationship between the α-component of acute survival curves and the average number of dsb induced by Gy and by DNA unit (200 Mbp).

(possibly because of [$^{14}$C]thymidine labelling, lysis buffer and electric field), and this value was slightly different for each cell type and for each experiment performed.

### Discussion

It has been realised for some time that human tumour cell lines can differ widely in their survival characteristics after treatment with ionising radiation. These differences are perhaps most marked when comparing cells of different histological types (Steel et al., 1989). However, within a single histological type there may also be significant variation in radiosensitivity, e.g. cervical carcinoma (Kelland & Steel, 1988). In terms of cellular response, the human breast and bladder cancer cell lines used in this study of cell survival and DNA damage after irradiation at high dose rates are representative of the central part of the range of radiosensitivities commonly seen in human tumour cell lines ($0.3 < SF2 < 0.7$).

In the light of claims by us and others that the level of initially induced DNA damage, rather than the damage after repair has taken place, may be a significant determinant of radiation sensitivity, this study has examined this parameter in these breast and bladder carcinoma cell lines. The data presented here (Figure 2a) show that only the T47D-B1 and RT-112 cell lines had a different level of induced damage when the slope of the DNA extraction curve was used as the damage end point. This is emphasised in Figure 2b, in which there is clearly no consistent relationship between SF2 and the slope of the DNA extraction curve. T47D-B1 has the steepest DNA extraction curve and yet is one of the most radioresistant breast cancer cell line.

If we examine the amounts of DNA extracted from the well and the shape of the distribution of DNA fragments in the gel lane (Figure 3a), we see a different picture. Figure 4a shows a wider spread of DNA damage induction curves and the relationship between damage induction measured in this way and radiosensitivity is much clearer. The relationship between the mean value of dsb induced per Gy and DNA unit in each cell line (slopes of the lines in Figure 4a) and its corresponding SF2 value is statistically significant (Figure 4b, $r = 0.955$, $P = 0.0031$). As expected from the nature of SF2 and the α-coefficient of the cell survival curve, there is also a close relationship between the α-coefficient and damage expressed in this way ($r = 0.943$, $P = 0.0049$) (Figure 4c). It has been suggested that the α-coefficient is a reflection of irreparable lesions, so the above relationship suggests that these may be a constant proportion of the induced lesions.

There are obvious inconsistencies in the data presented here, both in terms of assessing the role of the level of damage induction in the determination of radiosensitivity and in the way of expressing the extent of DNA fragmentation in PFGE experiments. We have shown in other studies that there is a good relationship between the slope of the DNA extraction curve in PFGE and SF2 among other cell lines of differing sensitivities (S.J. Whitaker & T.J. McMillan, submitted). The main difference between these studies and the current one is that the range of sensitivities was much greater in our previous study. In fact, the majority of the data presented here fall fairly well around the regression line seen in our previous, larger, study. We can therefore conclude that this technique may not be adequate for distinguishing cells which differ in sensitivity by only a small degree. The main deviation comes with T47D-B1, which appears to have a large degree of damage but is on the resistant site of the range of cell lines as judged by clonogenic cell survival. It is possible that this high degree of damage is compensated by a high ability to repair dsb, and this is currently under investigation.

The other inconsistency is between the two methods for assessing dsb levels using the same PFGE experiments. The ranking of the level of damage induced is different for the two analysis methods. It is not clear why this might be. One possibility is that the shape of the distribution of fragment sizes may not be the same for every cell line. Indeed, a comparison of the distribution of fragment sizes for the same total amount of DNA extracted for different cell lines does suggest that the distributions are not the same in each case (data not shown). There is currently some interest in the possibility of variation in susceptibility of different parts of the genome to damage (Chiu *et al.*, 1989). This may be related to chromatin structure, in which case the cell lines used in this study may prove to be interesting models to investigate this phenomenon.

Overall, these data suggest that within the small range of radiosensitivities in these cell lines the slope of the DNA extraction curve is not directly related to radiosensitivity. By taking into account the distribution of fragment sizes, however the level of induction does correlate with sensitivity. This demonstrates the power of PFGE in analysing strand breakage following treatment with ionising radiation and suggests lines of investigation into the significance of the distribution of fragment sizes in irradiated cells.

This work was supported by the Comisión Interministerial de Ciencia y Tecnologia (J.M.R.A., M.I.N., N.O., M.V. and V.P.) (CICYT SAL 89-1115 and DGICYT HB-250, Spain), (M.I.N. by PN90 26206833). T.J.M., C.M. and G.G.S. were supported by the Cancer Research Campaign, UK. A grant from the British Council greatly aided this collaboration.

### References

CHIU, S.M., FRIEDMAN, L.R. & OLEINICK, N.L. (1989). Formation and repair of DNA-protein crosslinks in newly replicated DNA. *Radiat. Res.*, **120**, 545–551.

CONTOPOULOU, C.R., COOK, V.E. & MORTIMER, R.K. (1987). Analysis of DNA double strand breakage and repair using orthogonal field alternation gel electrophoresis. *Yeast*, **3**, 71–76.

COOK, V.E. & MORTIMER, R.K. (1991). A quantitative model of DNA fragments generated by ionizing radiation, and possible experimental applications. *Radiat. Res.*, **125**, 102–106.

DEACON, J., PECKMAN, M.J. & STEEL, G.G. (1984). The radioresponsiveness of human tumours and the initial slope of the survival curves. *Radiother. Oncol.*, **2**, 317–323.

DEL MORAL, R., RUIZ DE ALMODÓVAR, J.M., FERNÁNDEZ, J.C., LOPEZ-GONZALEZ, J.D., VILLALBA, J., OLEA, N. & PEDRAZA, V. (1990). Relationship between proliferative activity and cellular hormonodependence in the MCF-7 breast cancer cell line. *Rev. Esp. Fisiol.*, **46**, 247–254.

FERTIL, B. & MALAISE, E.P. (1981). Inherent radiosensitivity as a basic concept for human tumour radiotherapy. *Int. J. Radiat. Oncol. Biol. Phys.*, **7**, 621–629.

ILLIAKIS, G. & OKAYASU, R. (1988). The level of induced DNA double-strand breaks does not correlate with cell killing in X-irradiated mitotic and G1 phase CHO cells. *Int. J. Radiat. Biol.*, **53**, 395–404.

KELLAND, L.R. & STEEL, G.G. (1988). Differences in radiation response among human cervix carcinoma cell lines. *Radiother. Oncol.*, **13**, 225–232.

KEYDAR, J., CHEN, L., KARBY, S.D., WEISS, F.R., DELAREA, J., RADU, M., CHAITCIK, S. & BRENNER, M.J. (1970). Establishment and characterization of a cell line of human breast carcinoma origin. *Eur. J. Cancer*, **15**, 659–670.

LIPPMAN, M., BOLAN, G. & HUFF, K. (1976). The effects of estrogen and antiestrogens on hormone-responsive human breast cancer in long-term tissue culture. *Cancer Res.*, **36**, 4595–4601.

MASTERS, J.R.W., HEPBURN, P.J., WALKER, L., HIGHMAN, W.J., TREJDOSIEWICZ, L.K., POVEY, S., HILL, B.T., RIDDLE, P.R. & FRANKS, L.M. (1986). Tissue culture models of transitional cell carcinoma: characterization of 22 human urothelial cell lines. *Cancer Res.*, **4**, 3630–3636.

OLIVE, P.L. (1992). DNA organization affects cellular radiosensitivity and detection of initial DNA strand breaks. *Int. J. Radiat. Biol.*, **62**, 389–396.

PEACOCK, J.H., EADY, J.J., EDWARDS, S.M., MCMILLAN, T.J. & STEEL, G.G. (1992). The intrinsic α/β ratio for tumour cells: is it a constant? *Int. J. Radiat. Biol.*, **61**, 479–487.

RADFORD, I.R. (1986). Evidence for a general relationship between the induced level of DNA double strand breakage and cell killing after X-irradiation of mammalian cells. *Int. J. Radiat. Biol.*, **49**, 611–620.

RUIZ DE ALMODÓVAR, J.M., MCMILLAN, T.J., OLEA, N., PEACOCK, J.H., PEDRAZA, V., STEEL, G.G., VILLALOBOS, M. & WHITAKER, S.J. (1993). Aspectos moleculares, celulares y multicelulares de la radiosensibilidad, In *Avances de la Investigacion Oncologica Española*, Lacal, J.C. & Barbacid, M. (eds), pp. 187–226. Madrid: Farmindustria Serie Científica.

SCHWARTZ, J.L. & VAUGHAN, A.T.M. (1989). Association among DNA/chromosome break rejoining rates, chromatin structure alterations and radiation sensitivity in human tumour cell lines. *Cancer Res.*, **49**, 5054–5057.

SOTO, A.M. & SONNENSCHEIN, C. (1985). The role of estrogens on the proliferation of human breast tumour cells (MCF-7). *J. Steroid Biochem.*, **23**, 87–94.

SOTO, A.M., MURAI, J.T., SITERI, P.K. & SONNENSCHEIN, C. (1986). Control of cell proliferation: evidence for negative control on estrogen-sensitive T47D human breast cancer cells. *Cancer Res.*, **46,** 2271–2275.

SOULE, D., VAZQUEZ, J., LONG, A., ALBERT, S. & BRENNAN, M. (1973). Human cell line from a pleural effusion derived from a breast carcinoma. *J. Natl Cancer Inst.*, **51,** 1409–1413.

STEEL, G.G., MCMILLAN, T.J. & PEACOCK, J.H. (1989). The picture has changed in the 1980s. *Int. J. Radiat. Biol.*, **56,** 525–537.

WHITAKER, S.J. & MCMILLAN, T.J. (1992). Oxygen effect for DNA double-strand break induction determined by pulsed-field gel electrophoresis. *Int. J. Radiat. Biol.*, **61,** 29–41.